# Influence of Habitat and Effects of Salt Stress on Biochemical and Physiological Parameters of *Glycyrrhiza uralensis*

**DOI:** 10.3390/plants13152108

**Published:** 2024-07-30

**Authors:** Junjun Gu, Tingting Jia, Miao Ma

**Affiliations:** Ministry of Education Key Laboratory of Xinjiang Plant Medicinal Resources Utilization, College of Life Sciences, Shihezi University, Shihezi 832003, China; gujunjun0120@163.com (J.G.); 20202006046@stu.shzu.edu.cn (T.J.)

**Keywords:** licorice, seedlings, salt stress, active oxygen, chlorophyll fluorescence, photosynthetic gas exchange

## Abstract

The seeds of *Glycyrrhiza uralensis* Fisch. used for cultivating are primarily sourced from wild populations. However, the types of habitats where wild *G. uralensis* grow are diverse. We studied the effects of salinity on the growth, antioxidant capacity, and photosynthetic physiology of two-month-old licorice seedlings from different habitats to evaluate their salt tolerance. With the increasing NaCl concentration, compared with non-salinized habitats, seedlings originating from seeds collected from salinized habitats showed milder inhibition in root biomass and root volume. Also, the crown diameter increased more significantly. Activities of superoxide dismutase, catalase, and peroxidase are higher. Correspondingly, the electrolyte leakage rate of the leaves is low. Their leaves had a higher photoprotection capacity and potential maximum photochemical efficiency of PSII. Net photosynthetic rate, transpiration rate, and stomatal conductance showed less inhibition under 4 and 6 g/kg NaCl treatment. The content of glycyrrhizic acid and glycyrrhetinic acid in their roots was significantly increased under 2 g/kg NaCl treatment and was significantly higher than that of seedlings from non-salinized habitats under the same NaCl treatment. In conclusion, seeds from salinized habitats show improved tolerance to salt stress at the seedling stage, which is attributed to their superior phenotypic adaptability, strong antioxidant, and especially high light protection ability.

## 1. Introduction

*Glycyrrhiza uralensis* Fisch. is a herb of Leguminosae, predominantly growing in semi-arid desert areas of Asia, Europe, and North America [1]. It features a large root system and extensive plant coverage, making it instrumental in the restoration of sandy grassland ecosystems [2]. The dry roots and rhizomes of licorice have the characteristics of anti-inflammatory, antitussive, and reconciling, making it a traditional Chinese medicine [3]. Secondly, they are rich in glycyrrhizic acid, glycyrrhetinic acid and flavonoids, which have many pharmacological effects such as antiviral [4], antibacterial [5], nerve protection [6], anti-breast cancer [7], improvement of atopic dermatitis [8], inhibition of muscle fibrosis [9] and improving prostatic hyperplasia [10]. In addition, they are rich in natural sweetening compounds, including licorice polysaccharides, glycyrrhetinic acid, and glycyrrhetinic acid, making them an excellent additive for enhancing the flavor of candies and chewing gum [11]. A growing market demand coupled with a significant decline in wild resources has spurred the cultivation of licorice [12]. 

Cultivated licorice is often planted in salinized soil, resulting in the development of seedlings with poor vigor, which will inevitably affect the plant’s growth and development in later stages [13,14]. Therefore, it is imperative to enhance the salt tolerance of licorice seedlings. Wild *G. uralensis* thrives in a variety of habitats, capable of growing and completing its life cycle in soils with total salt content ranging from 0 to 0.7% [15,16]. Previous studies have found that the salt tolerance of plants growing in a saline environment for a long time is enhanced [17,18], which may be the result of natural selection leading to the retention of salt-tolerant individuals or salt-mediated maternal environmental effects promoting the salt tolerance of plants. In previous studies, we found that the offspring seeds of *G. uralensis* from saline habitats have stronger salt tolerance under salt stress compared to non-saline habitats [19]. However, the salt tolerance of *G. uralensis* from different habitats may differ between germination and seedling stages. We assume that *G. uralensis* inhabiting salt habitats helps them to have better adaptability under salt stress; is this assumption valid? At present, there are no relevant research reports.

Salt stress causes osmotic, ionic, and oxidative stress, increasing the accumulation of toxic substances in plants, destroying the permeability of the plant cell membrane system, and then affecting the growth and development of plants, including inhibiting the plant height, crown width, basal stem, leaf number, root volume, and root biomass of seedlings [20]. Photosynthesis, as the most basic activity for maintaining plant life, is most sensitive to salt stress during the growth process, and its changes can be used to determine changes in plant vitality [21]. Compared with the “apparent” gas exchange parameters, chlorophyll fluorescence parameters have the characteristic of reflecting the “intrinsic” nature of plants due to their close correlation with various processes in light reactions [22]. By combining chlorophyll fluorescence analysis with gas exchange parameters, the response mechanism of the *G. uralensis* photosynthetic system to salt stress can be understood more comprehensively. 

Therefore, this study uses the seeds of *G. uralensis* from saline and non-saline soil habitats as experimental materials. Under different concentrations of NaCl treatment, by comparing their growth indicators, biomass accumulation, and antioxidant capacity, the differences in salt tolerance capabilities during the seedlings stage were evaluated. Furthermore, through photosynthetic gas exchange parameters, chlorophyll fluorescence parameters, and the plasticity responses of various growth indicators, the reasons for the differences in salt tolerance between the two were analyzed. In addition, as *G. uralensis* is a medicinal plant, we also measured the content of glycyrrhizic acid and glycyrrhetinic acid in the roots of both plants to characterize the quality of licorice as a medicinal material, aiming to provide experimental evidence for the selection of salt-tolerant *G. uralensis* plants.

## 2. Results

### 2.1. Growth Indexes of the Seedlings

**Figure 1:**
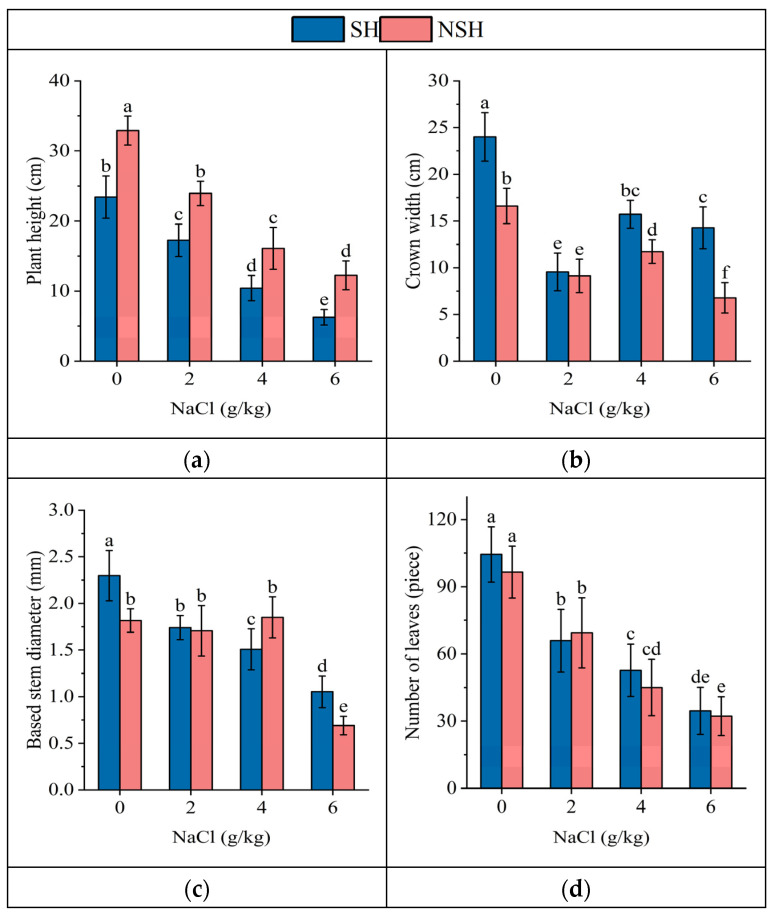

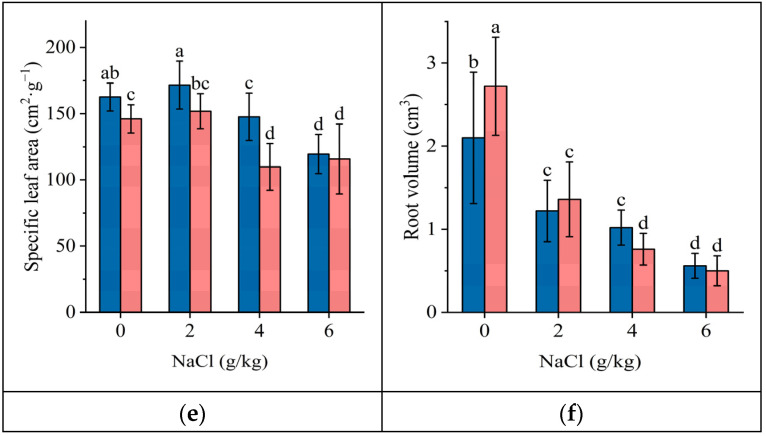
Effects of NaCl treatments (0, 2, 4 and 6 g/kg) on plant height (**a**), crown width (**b**), based stem diameter (**c**), number of leaves (**d**), specific leaf area (**e**), and root volume (**f**) of *G. uralensis* seedlings from SH and NSH. Value represents the mean of 10 replicates ± standard deviation, *p*-value < 0.05. (Different letters above the bars indicate significant differences based on Duncan’s test. SH represents salinized habitat, NSH represents non-salinized habitat).

The habitat and salt treatment and their interaction had significant effects on the plant height, crown width, based stem diameter, and specific leaf area. NaCl treatment significantly inhibited the plant height, crown width, based stem diameter, and specific leaf area of licorice seedlings (Table 1). The plant height of seedlings originating from seeds collected from the NSH was significantly higher than that of seedlings originating from seeds collected from the SH under different salt treatments. The specific leaf area of seedlings originating from seeds collected from the SH was significantly larger than that of seedlings originating from seeds collected from NSH under control, mild, and moderate NaCl treatments. Under moderate and severe NaCl treatments, the crown width of seedlings originating from seeds collected from the SH was significantly larger than that of seedlings originating from seeds collected from NSH, which was 1.3 and 2.1 times that of seedlings originating from seeds collected from the NSH. The root volume of seedlings originating from seeds collected from the SH was significantly higher than that of seedlings originating from seeds collected from the NSH under moderate NaCl stress. Overall with the increasing NaCl treatment, the crown width of seedlings decreased first and then increased, and the based stem diameter and root volume decreased gradually. However, the crown width of seedlings originating from seeds collected from the SH increased more significantly, the based stem diameter decreased more significantly, and the root volume decreased more slowly (Figure 1).

### 2.2. Biomass Accumulation of the Seedlings

**Figure 2:**
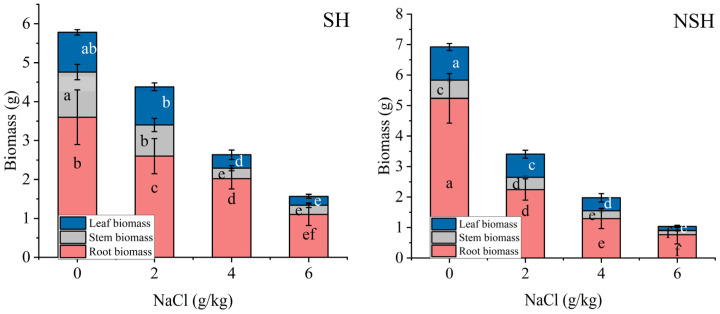
Effect of NaCl (0, 2, 4 and 6 g/kg) on the root, stem, and leaf biomass of *G. uralensis* seedlings from SH and NSH. Value represents the mean of 10 replicates ± standard deviation, *p*-value < 0.05. (Different letters above the bars indicate significant differences based on Duncan’s test. SH represents salinized habitat, NSH represents non-salinized habitat).

Salt treatment and the interaction between the salt treatment and habitat had a significant effect on the root, stem, and leaf biomass, while the habitat only had a significant effect on the stem biomass. NaCl treatment significantly inhibited the root, stem, and leaf biomass of licorice seedlings (Table 2). The stem biomass of seedlings originating from seeds collected from the SH was significantly higher than that of seedlings originated from seeds collected from the NSH under control, mild, and severe stress conditions, which was 2.0, 2.0, and 1.8 times higher than that of seedlings originating from seeds collected from the NSH, respectively. The leaf biomass and root biomass of seedlings originating from seeds collected from the NSH seedlings in the control group were the largest. The decrease in leaf biomass and root biomass with the increase in NaCl concentration was greater than that of individuals originating from seeds collected from the SH. Under mild and moderate NaCl treatments, the root biomass of seedlings originating from seeds collected from the NSH was 82.8% and 62.5% of the root biomass of seedlings originating from seeds collected from the SH, respectively. Under mild NaCl treatment, the leaf biomass of seedlings originating from seeds collected from the NSH was 77.8% of the leaf biomass of seedlings originating from seeds collected from the SH (Figure 2). 

### 2.3. Antioxidant Capacity of the Seedlings

**Figure 3:**
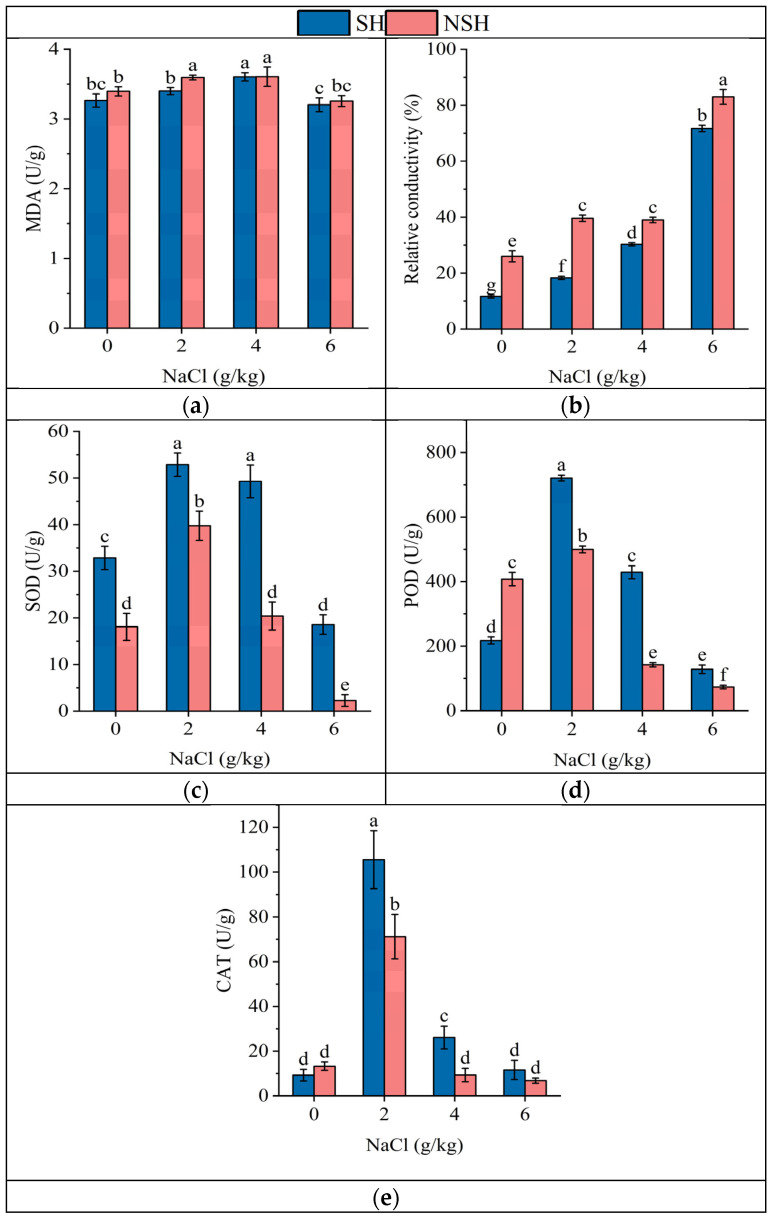
Effect of NaCl (0, 2, 4 and 6 g/kg) on malondialdehyde (**a**), relative conductivity (**b**), superoxide dismutase (**c**), peroxidase (**d**), and catalase (**e**) in leaves of *G. uralensis* from SH and NSH. Value represents the mean of 3 replicates ± standard deviation, *p*-value < 0.05. (Different letters above the bars indicate significant differences based on Duncan’s test. SH represents salinized habitat, NSH represents non-salinized habitat).

The habitat and salt treatment and their interaction had significant effects on SOD, POD, CAT and REC (Table 3). With the increasing NaCl concentration, the relative electrolyte leakage rate continued to increase, and the content of MDA and the activity of antioxidant enzymes in leaves increased first and then decreased. Under mild NaCl stress conditions, the MDA content of seedlings originating from seeds collected from the SH was significantly lower than that of seedlings originating from seeds collected from the NSH. The relative electrolyte leakage of seedlings originating from seeds collected from the SH was significantly lower than that of seedlings originating from seeds collected from the NSH under each treatment, which were 43.7%, 46.3%, 78.8%, and 85.8% of the NSH, respectively. Compared with the NSH, the activities of SOD, CAT and POD in seedlings originating from seeds collected from the SH were significantly higher than those in seedlings originating from seeds collected from the NSH at different NaCl concentrations (Figure 3).

### 2.4. Photosynthetic Parameters of the Seedlings

**Figure 4:**
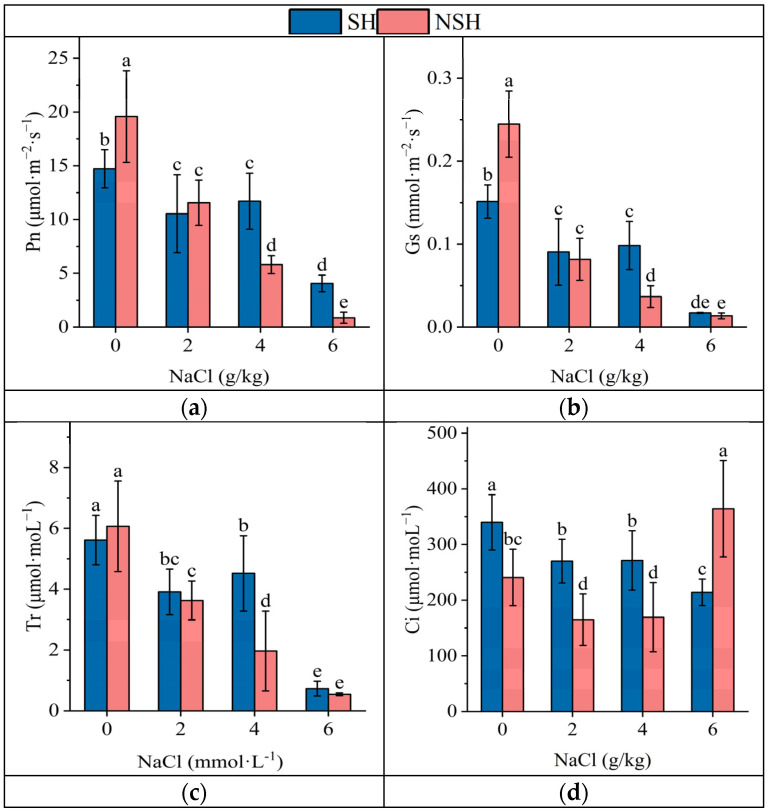
The effect of NaCl (0, 2, 4 and 6 g/kg) on the net photosynthetic rate (**a**), stomatal conductance (**b**), transpiration rate (**c**), and intercellular carbon dioxide concentration (**d**) of *G. uralensis* seedlings from SH and NSH. Value represents the mean of 12 replicates ± standard deviation, *p*-value < 0.05. (Different letters above the bars indicate significant differences based on Duncan’s test. SH represents salinized habitat, NSH represents non-salinized habitat).

The salt treatment and the interaction between the salt treatment and habitat had significant effects on Pn, Tr, Ci ,and Gs, while the habitat only had significant effects on Tr and Ci. The salt treatment significantly inhibited the Pn, Tr and, Gs of licorice seedlings (Table 4). In the control group, the Pn, Tr, and Ci of seedlings originating from seeds collected from the NSH were the largest. With the increase in NaCl treatment concentration, the Pn, Tr, and Gs of licorice seedlings from different sources gradually decreased, and the decrease in seedlings originating from seeds collected from the NSH was greater. The Pn, Tr, and Gs of seedlings originating from seeds collected from the SH were significantly higher than those of seedlings originating from seeds collected from the NSH under moderate salt stress, which were 2, 2.3, and 2.7 times higher than those of seedlings originating from seeds collected from the NSH, respectively. The Pn of seedlings originating from seeds collected from the SH and NSH were 4 and 0.9 μmol·m^−2^·s^−2^ under severe NaCl stress, respectively; the Pn of seedlings originating from seeds collected from the SH was still obviously higher than that of seedlings originating from seeds collected from the NSH. The Ci of seedlings originating from seeds collected from the SH gradually decreased with the increasing NaCl concentration, while the Ci of seedlings originating from seeds collected from the NSH decreased first and then increased. Under different concentrations of NaCl treatment, the Ci of seedlings originating from seeds collected from the SH were 140%, 160%, 168%, and 58.7% of the seedlings originating from seeds collected from the NSH, respectively (Figure 4).

### 2.5. Fluorescence Parameters of the Seedlings

**Figure 5:**
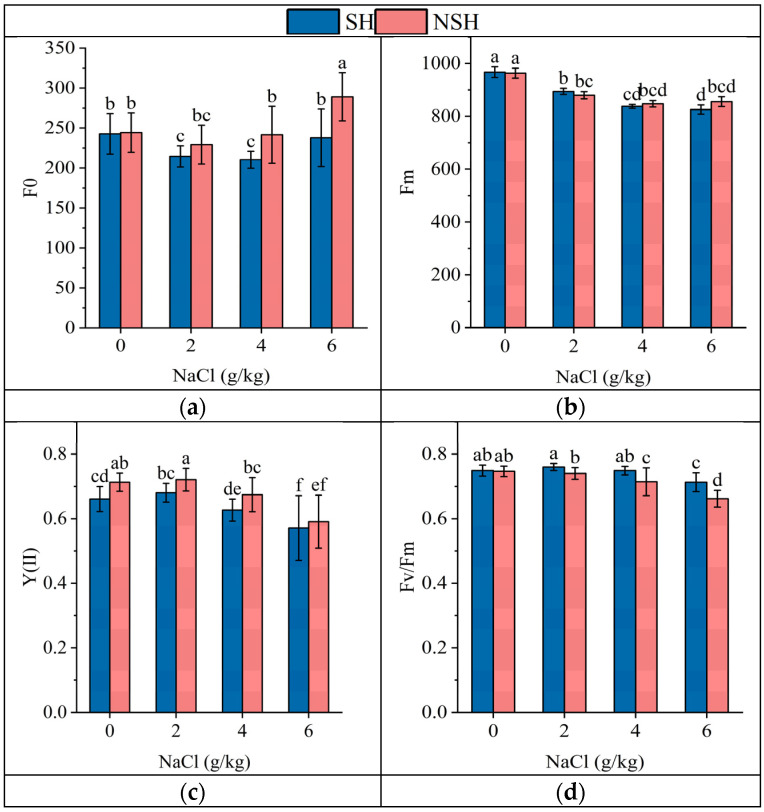
Effects of NaCl (0, 2, 4 and 6 g/kg) on initial fluorescence (**a**), maximum fluorescence (**b**), potential maximum photosynthetic efficiency (**c**), and actual photosynthetic efficiency (**d**) in leaves of *G. uralensis* seedlings from SH and NSH. Value represents the mean of 15 replicates ± standard deviation, *p*-value < 0.05. (Different letters above the bars indicate significant differences based on Duncan’s test. SH represents salinized habitat, NSH represents non-salinized habitat).

The habitat and salt treatment had significant effects on F_0_, F_v_/F_m_, YII, ΦPsII, Y(NO), and Y(NPQ), and their interaction only had significant effects on F_0_ and F_v_/F_m_ (Table 4). With the increase in NaCl concentration, F_v_/F_m_, YII, ΦpsII, and Y(NO) decreased significantly, but F_0_ and Y(NPQ) increased gradually. The F_0_ of seedlings originating from seeds collected from the SH was significantly lower than that of seedlings originating from seeds collected from the NSH in each NaCl treatment group, which was 93.4%, 87.1%, and 82.2% of seedlings originating from seeds collected from the NSH, respectively. With the increase in NaCl concentration, the F_v_/F_m_ and YII of the two populations decreased gradually. It is worth noting that the F_v_/F_m_ of seedlings originating from seeds collected from the NSH was lower than that of seedlings originating from seeds collected from the SH under the same NaCl treatment. The F_v_/F_m_ of seedlings originating from seeds collected from the NSH was 97%, 95.4%, and 92.7% of the F_v_/F_m_ of the seedlings originating from seeds collected from the SH, respectively. However, the YII of seedlings originating from seeds collected from the NSH was higher than that of seedlings originating from seeds collected from the SH, which was 1.07, 1.06, and 1.07 times higher than that of seedlings originating from seeds collected from the SH under the control, mild, and moderate NaCl treatments, respectively (Figure 5). 

**Figure 6:**
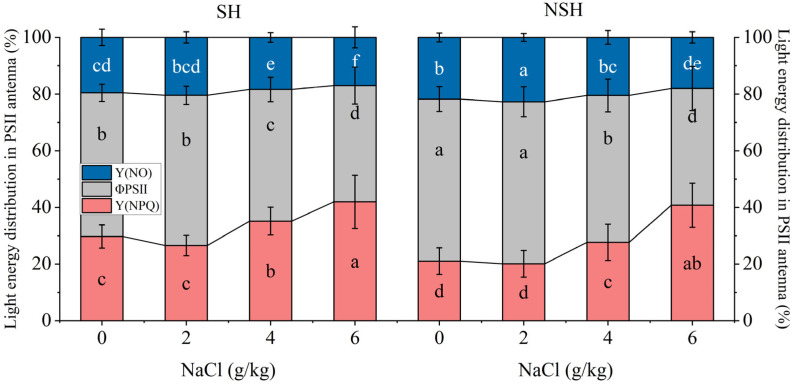
Effect of NaCl (0, 2, 4 and 6 g/kg) on proportion of the light energy absorbed in PSII antenna that is dissipated thermally Y(NPQ), used in photochemistry ΦpsII, and the remaining light energy excess Y(NO) in leaves of *G. uralensis* seedlings from SH and NSH. Value represents the mean of 15 replicates ± standard deviation, *p*-value < 0.05. (Different letters above the bars indicate significant differences based on Duncan’s test. SH represents salinized habitat, NSH represents non-salinized habitat).

The habitat and salt treatment had significant effects on ΦPsII, Y(NO), and Y(NPQ) and the interaction between the two had no significant effect on them (Table 4). The PII of seedlings originating from seeds collected from the SH and NSH increased Y(NPQ) and decreased ΦpsII with the increasing NaCl concentration. Compared with seedlings originating from seeds collected from the NSH, the PSII of seedlings originating from seeds collected from the SH accounted for a higher proportion of Y(NPQ) and a lower proportion of Y(NO) in each treatment (Figure 6). 

### 2.6. Contents of Secondary Metabolites of the Seedlings

**Figure 7:**
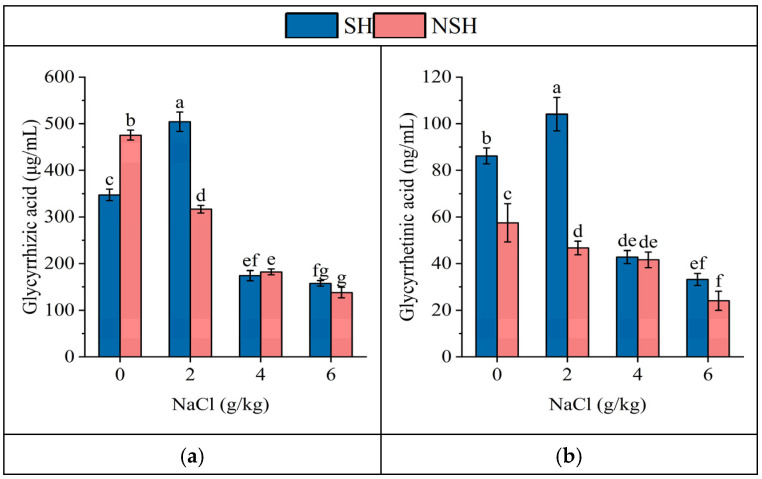
Effect of NaCl (0, 2, 4 and 6 g/kg) on the content of glycyrrhizic acid (**a**) and glycyrrhetinic (**b**) acid in the roots of *G. uralensis* seedlings from SH and NSH. Value represents the mean of 3 replicates ± standard deviation, *p*-value < 0.05. (Different letters above the bars indicate significant differences based on Duncan’s test. SH represents salinized habitat, NSH represents non-salinized habitat).

The habitat and salt treatment and their interaction had significant effects on glycyrrhizic acid and glycyrrhetinic acid (Table 2). The contents of glycyrrhizic acid and glycyrrhetinic acid in the roots of seedlings originating from seeds collected from the NSH decreased gradually with the increase in NaCl concentration. But the contents of two medicinal components of seedlings originating from seeds collected from the SH increased first and then decreased, and reached the maximum value of 504.3 μg/mL and 104.3 ng/mL in the mild salt stress, which were 1.61 and 2.26 times that of seedlings originating from seeds collected from the NSH, respectively (Figure 7).

## 3. Discussion

The maternal environmental effect refers to the influence of the environmental conditions of the mother plant on the performance and characteristics of its offspring [23]; it is also the phenotypic response of the offspring to environmental heterogeneity [24]. Among the different ecotypes of many plants, such as *Campanula glomerata* [25], *Triticum aestivum* [26], and *Suaeda aegyptiaca* [27], the maternal environmental effects have been extensively studied. Will the salt tolerance of *G. uralensis* be different due to the maternal habitat? Compared with the seedlings originating from seeds collected from the NSH, the total biomass of seedlings originating from seeds collected from the SH was significantly higher in each NaCl treatment group. Seedlings from the NSH were more sensitive to NaCl. For example, NaCl stress exerted more significant inhibitory effects on Pn, Tr, Gs, F_v_/F_m_, Y(II), ΦPsII, the antioxidant capacity of leaves, individual growth and the root biomass; in other words, seedlings of *G. uralensis* from the SH were more salt-tolerant. 

Biomass is the most direct indicator of plant resistance and root biomass also determines the medicinal materials yield of *G. uralensis*. The plasticity of plant growth and biomass allocation is one of the survival strategies of plants under adversity [28]. Compared with the NSH, the total biomass of *Ipomoea cairica* [29] and *Medicago truncatula* [30] seedlings from salinized habitats was higher under salt stress, which was consistent with our results, but opposite to the study of *Solidago canadensis* [31]. This indicates that the accumulation pattern of biomass of the same plant from salinized and non-salinized habitats will vary depending on the type of plant. With the increase in salt concentration, the plant height, crown width, leaf number, based stem diameter, and root volume of *G. uralensis* in different habitats decreased, but the crown diameter increased under moderate and severe salt stress, which may increase the effective photosynthetic area of the plant to improve its photosynthetic capacity. In contrast, the seedlings originating from seeds collected from the SH had a more significant regulation of the crown width. Secondly, with the increase in the salt stress, seedlings originating from seeds collected from the SH had a larger leaf area, slower root volume decrease, and faster stem diameter decrease, which may be an important reason for its higher biomass accumulation. In addition, there is a significant difference in plant height between the two under different treatments, with seedlings from the SH being shorter, which may be the result of their long-term adaptation to the local environment. Therefore, *G. uralensis* seedlings originating from seeds collected from the SH have a higher total biomass and root biomass due to their stronger phenotypic plasticity. In conclusion, *G. uralensis* from the NSP had higher fitness just in non-saline soil, while *G. uralensis* from the SH had higher fitness in saline soil, which means that the salinity-mediated maternal effect improves the salt tolerance of *G. uralensis*.

Salt stress often causes an increase in reactive oxygen species in plants, which destroys the selective permeability of the membrane and increases the accumulation of toxic substances. Reactive oxygen species are often catalyzed by SOD enzymes to produce hydrogen peroxide, and then catalyzed by CAT and POD enzymes to produce water and oxygen [32]. Although seedlings under mild NaCl stress significantly enhanced the activities of antioxidant enzymes such as SOD, CAT and POD, the relative electrolyte leakage of leaves was significantly higher than that of the control group, indicating that antioxidant enzymes could not effectively alleviate the oxidative damage caused by salt, especially the seedlings originating from seeds collected from the NSH. *Zygophyllum xanthoxylum* [33] from salinized habitats showed higher SOD activity than those from non-salinized habitats under 200 and 400 mmol/L NaCl treatments. When studying the salt tolerance of *Zygophyllum coccineum* [34] in different habitats, it was also found that the higher the soil salinity in the mother habitat was, the higher was the activity of SOD enzymes in seedlings, which is consistent with our results. Overall, the activities of SOD, POD, and CAT of seedlings originating from seeds collected from the SH were much higher than those of seedlings originating from seeds collected from the NSH, which is why licorice seedlings originating from seeds collected from the SH had lower oxidative damage and less electrolyte leakage, which may be the result of their long-term adaptation to a high-salt habitat.

Photosynthesis is the basis of plant growth, development, and morphogenesis, and it is also one of the main processes affected by salt stress [35]. The decrease in photosynthetic capacity under salt stress conditions is often due to stomatal closure caused by osmotic stress [36], which leads to insufficient absorption of CO_2_, resulting in a reduction in the net photosynthetic rate. The Gs and Tr of seedlings originating from seeds collected from the NSH decreased more significantly under moderate NaCl treatment than that of mild NaCl stress, but seedlings originating from seeds collected from the SH did not decrease, indicating that seedlings originating from seeds collected from the SH had higher tolerance to osmotic stress induced by moderate NaCl, which may be the result for its greater root biomass under moderate NaCl treatment. In addition, the variation in patterns of Ci and Gs of seedlings originating from seeds collected from the NSH was different with the increasing NaCl concentration; that is, Gs decreased significantly, while Ci increased gradually. In other words, when the degree of stomatal closure is higher, the intercellular CO_2_ concentration is higher, which means that the utilization rate of CO_2_ in the leaves may decrease. This indicated that the decline in Pn of seedlings originating from seeds collected from the NSH under moderate salt stress was due to other reasons besides stomatal limitation [21].

After the antenna pigment captures light energy, a small part of them is emitted again in the form of light, namely fluorescence, its generation and quenching of which can accurately reflect the absorption, transmission, dissipation, and distribution of light energy in the light system [37]. In order to further analyze the difference in photosynthetic activity between the seedlings from the SH and NSH under NaCl stress, we compared the chlorophyll fluorescence parameters of the two. The F_0_ is determined by the characteristics of the plant itself; F_v_/F_m_ represents the theoretical maximum light energy conversion efficiency of PSII. Y(II) is the actual light energy conversion efficiency of PSII [38]. The F_0_ of seedlings originating from seeds collected from the NSH increased more significantly than those of seedlings originating from seeds collected from the SH under severe NaCl stress, which meant that the permeability of thylakoid membrane changed, and the chlorophyll in thylakoid leaked, resulting in the enhancement of background fluorescence. The F_v_/F_m_ of seedlings originating from seeds collected from the SH and NSH decreased significantly under severe and moderate salt stress conditions, respectively, indicating that the activity of PSII of seedlings originating from seeds collected from the NSH was more vulnerable to salt stress. This also means that the significant decrease in Pn is related to the significant decrease in the activity of photoreaction centers in addition to stomatal factors [21]. Excessive energy accumulated in mesophyll cells will damage the activity of photoreaction centers under NaCl stress. On the one hand, higher Y(NPQ) indicates that a plant receives an excess of light intensity, and on the other hand, that the plant can still protect the light response center through self-regulation (such as dissipating excess light energy into heat) [39]. Higher Y(NO) indicates that the mechanism of photochemical energy conversion and protective regulation (heat dissipation) is not sufficient to completely consume the light energy absorbed by plants. *G. uralensis* from different habitats can dissipate excess light energy into heat to protect the light reaction center by increasing Y(NPQ) and reducing Y(NO) under NaCl stress, seedlings originating from seeds collected from the SH had a higher proportion of excessive energy dissipation into heat and a lower proportion of fluorescence emission which indicates that the PSII of seedlings originating from seeds collected from the SH has higher light protection ability. Under salt stress, the Y(II) of seedings originating from seeds collected from the NSH was significantly higher than that of seedlings originating from seeds collected from the SH, but its Pn was lower than that of the latter, which is the same as the relevant reports on *Oenothera drummondii* [40]. For the imbalance in photochemical reaction and CO_2_ assimilation between seedlings originating from seeds collected from the SH and NSH, we infer that the photosynthetic efficiency of seedlings originating from seeds collected from the NSH with higher actual light energy conversion efficiency was reduced, and its electrons reduced by Qp protein may flow to other electron-consuming physiological processes, such as photorespiration. In view of the differences in net photosynthetic rate and total biomass between the two, we believe that seedlings originating from seeds collected from the SH have a better energy distribution strategy for salt stress.

The medicinal efficacy and sweetness of licorice roots mainly come from glycyrrhizic acid and glycyrrhetinic acid [41]. In our study, the accumulation of glycyrrhizic acid and glycyrrhetinic acid in the roots of seedlings originating from seeds collected from the NSH was significantly inhibited with the increasing NaCl concentration. However, the contents of the two components in the roots of seedlings originating from seeds collected from the SH were significantly increased under mild salt treatment (both were significantly higher than those seedlings originating from seeds collected from the NSH under the same salt treatment). In Xu’s study [42], the accumulation pattern of secondary metabolites in *Apocynum venetum* from different habitats under salt stress was consistent with our results, that is, under salt stress, the contents of quercetin and kaempferol in the leaves of *Apocynum venetum* from salinized habitats were significantly higher than those from non-salinized habitats.

## 4. Conclusions

In this study, the antioxidant capacity, growth, and biomass of *G. uralensis* seedlings originating from seeds collected from saline and non-saline habitats were significantly inhibited and reduced under salt stress, but *G. uralensis* originating from seeds collected from saline habitats shows improved tolerance to salt stress compared with those from non-salinized habitats. Compared with non-saline habitats, seedlings originating from seeds collected from saline habitats have higher photoprotective capacity under salt stress, resulting in higher photochemical activity and photosynthetic capacity under salt stress. In addition, the positive plasticity changes in its crown width and the larger specific leaf area played a positive role in the process of salt stress tolerance. For licorice seedlings in salinized habitats, mild NaCl stress contributed to the accumulation of glycyrrhizic acid and glycyrrhetinic acid in their roots, which provided a new way for the directional accumulation of secondary metabolites. Our results show that the salt tolerance of *G. uralensis* will be different due to the salinity of the parent habitat; that is, seedlings from salinized habitats show a greater degree of tolerance to salt stress, which is consistent with the salt tolerance pattern of the two during seed germination.

## 5. Materials and Methods

### 5.1. Materials

*G. uralensis* seeds were collected from Buerjin County, Xinjiang (N 47°54.9234′ E 86°50.2838′) in October 2021. The non-salinized habitat (NSH) is located in the middle reaches of a flood alluvial fan (edge of corn farmland, soil water-soluble total salt 0.4 ± 0.03 g/kg) previously discovered by the research group, and the salinized habitat (SH) is located downstream of the flood alluvial fan (salinized lowland meadow, soil water-soluble total salt 10.1 ± 1.2 g/kg). The voucher specimens of *G. uralensis* from saline and non-saline habitats were stored in the Institute of Licorice, Shihezi University. The specimen numbers were ”20210421” and ”20210422”, respectively. 

### 5.2. Experimental Design

Healthy and full seeds of *G. uralensis* from different habitats with the same size were broken-dormancy, disinfected, and germinated [19]. After 3 days of germination, four licorice seedlings exhibiting uniform growth were carefully selected and transplanted into a plastic pot (30 cm high, 23 cm in diameter) on 1 May 2022. The soil in the plastic pot was from the licorice field (pH 7.8, total nitrogen, phosphorus, and potassium concentrations were 0.315 g/kg, 0.131 g/kg and 5.47 g/kg, respectively, available nitrogen, phosphorus, and potassium levels were 52.59 mg/kg, 5.23 mg/kg and 50.04 mg/kg, respectively, and the organic matter content was 6.64 mg/kg). Each pot contained 8 kg of soil. After the seedlings grew to four true leaves (in about 1 month), they were treated with NaCl on 2 June 2022. Four salt concentration gradients were set, including 0 g/kg (control group), 2 g/kg (mild stress), 4 g/kg (moderate stress), and 6 g/kg (severe stress) according to the classification of salinized soil and the NaCl concentration range that *G. uralensis* could tolerate [43,44]. The actual amount of total NaCl added per pot was 0 g, 16 g, 32 g, and 48 g, respectively. After a week of salt treatment, the relevant indicators were measured. To avoid seedlings being burnt by high concentrations of salt, NaCl was applied multiple times, which was added every 3 days. A total of 0 g, 1.6 g, 3.2 g, and 4.8 g of NaCl was dissolved in 300 mL of water, and then the seedlings were irrigated so that each treatment reached the predetermined NaCl concentration at the same time after 10 irrigations. Each treatment planted five pots. During the experiment, 100 mL of water was replenished every morning and evening so that the relative soil moisture of the basin soil was maintained at 60%. The plastic pots were strategically positioned in an open area of the Shihezi University campus, ensuring that each pot was spaced more than 50 cm apart from its neighbors. To maintain uniform light exposure for the seedlings, the placement of the pots was shuffled randomly once every week.

### 5.3. Determination of Gas Exchange

Seven days following the conclusion of the salt treatments, on a clear day, the first fully expanded leaf from the apex of each seedling was chosen for the determination of photosynthetic gas exchange parameters and chlorophyll fluorescence parameters. 

Measurements of the instantaneous gas exchange indices of these leaves, including net photosynthetic rate (Pn), stomatal conductance (Gs), transpiration rate (Tr), and intercellular CO_2_ concentration (Ci), were conducted using the Li-6400 portable photosynthesis system (model Li-6400, Li-Cor Biosciences, Lincoln, NE, USA). The artificial light sources provided an illumination intensity of 1800 µmol m^−2^ s^−1^. Each treatment was replicated 12 times to ensure reliability of the results.

### 5.4. Determination of Chlorophyll Fluorescence Parameters

A multifunctional plant measuring instrument (MultispeQ V2, Yingchi Technology Co., Ltd., Beijing, China) was used to determine the minimum fluorescence under light (F_0_′), maximum fluorescence under light (F_m_′), variable fluorescence under light (F_v_′), steady-state fluorescence (F_s_), initial fluorescence (F_0_), maximum fluorescence (F_m_), and variable fluorescence (F_v_) of the leaves [45]. The potential maximum photosynthetic efficiency F_v_/F_m_, actual photosynthetic efficiency or actual quantum yield Y(II) [46], quantum yield of regulated energy dissipation Y(NPQ), and quantum yield of non-regulated energy dissipation Y(NO) were calculated using the following formula [47,48]. The light energy absorbed by PSII is dissipated in three fractions, the one absorbed by the PSII antennae used in photochemistry (ΦPsII = Y(II)), the one dissipated thermally Y(NPQ), and the remaining fraction, not going into either of them, known as excess Y(NO). Each treatment was repeated 15 times to ensure reliability of the results. The fluorescence parameters F_0_′, F_m_′, and F_s_ under light adaptation were measured in sunny cloudless or less cloudy weather. The test time was 9:00–12:00 Beijing time, and the fluorescence parameters F_0_, F_m_, and F_v_ under dark adaptation were measured after the plant was fully dark adapted for at least 20 min during the night from 22:30 to 24:00.

F_v_/F_m_ = (F_m_ − F_0_)/F_m_

Y(II) = (F_m_′ − F_S_)/F_m_′

NPQ = F_m_/F_m_′ − 1

qP = 1 − (F_s_ − F_0_′)/(F_m_′ − F_0_′)

qL = qP × F_0_′/F

Y(NPQ) = 1 − Y(II) − 1/(NPQ + 1 + qL(F_m_/F_0_ − 1))

Y(NO) = 1/(NPQ + 1 + qP(F_m_/F_0_ − 1))

ΦPsII + Y(NO) + Y(NPQ) = 1

### 5.5. Determination of Physiological Indexes

After randomly collecting 15 upper leaves of licorice seedlings in each treatment, they were quickly placed in an ice box. A total of 0.1 g of leaves were accurately weighed for the determination of the following physiological indicators. The antioxidant capacity of plants was evaluated by measuring the activities of superoxide dismutase (SOD), catalase (CAT), and peroxidase (POD) using the enzyme activity kit provided by the Soleibao Company [49]. To ascertain the genuine physiological condition of the leaves, their relative electrolyte leakage and malondialdehyde content were evaluated using a conductivity meter (DDS-11A, Shanghai Leici Laboratory Desktop Digital Conductivity Meter, China) and the thiobarbituric acid method [50], respectively. Each index for the treatment groups underwent three rounds of measurement to ensure accuracy and consistency.

### 5.6. Determination of Growth Indicators

On the 90th day after finishing the NaCl treatment (1 October 2022), we selected 10 seedlings from each treatment and measured their plant height, crown diameter, base stem diameter (stem thick), and leaf number per plant [51]. After carefully washing the soil off the root surface, an Epson Expression 11000XL scanner was used to measure the total root volume and total leaf area of each seedling. The leaves were then placed in an oven at 70 °C, and dried to a constant weight; we weighed the accurate mass of the leaves and calculated the specific leaf area of the leaves using the following formula with each treatment repeated three times [52].

Specific leaf area (cm^2^ × g^−1^) = leaf area (cm^2^)/leaf dry weight (g).

In addition, another 10 seedlings were selected from each treatment and we separated their roots, stems, and leaves, then dried them in an oven at 70 ℃ to a constant weight, tested their dry weights and calculated the mean value.

### 5.7. Determination of Medicinal Components of Roots

The UPLC-MS/MS analyses to determine the concentrations of glycyrrhizic acid and glycyrrhetinic acid were conducted using a Waters’ Acquity Ultra-Performance Liquid Chromatography System paired with an MS detector [53]. The dried roots of *G. uralensis* were mixed, crushed into powder, and passed through a 40-mesh sieve. A total of 0.100 g of powder in each treatment was weighed accurately and put into a 10 mL test tube, 7 mL of 60% ethanol was added, and the tube was placed into a 300W ultrasonic extractor (KQ-300E, Kunshan Ultrasonic Instrument Co., Ltd., Shanghai, China) to ultrasonically extract for 2 h, and then centrifuged with a refrigerated centrifuge (12,000 r/min) for 30 min; the supernatant liquor was obtained and filtered through a 0.25 μm microfiltration membrane. For each treatment, three samples were prepared, and each sample was analyzed in triplicate. The UPLC-MS/MS separations took place on a 50 mm × 2.1 mm UPLC BEH C18 column with a 1.7 μm particle size, at a flow rate of 0.3 mL/min, a column temperature of 30 °C, and an injection volume of 1 μL. The optimized mobile phase consisted of 0.1% formic acid in water (A) and acetonitrile (B), employing a gradient elution of 20–98% B over 0–3.0 min, maintaining 98% B from 3.0 to 4.5 min, switching back to 98–20% B from 4.5 to 5.0 min, and finally stabilizing at 20% B from 5.0 to 7.0 min.

The ion source was an electrospray ionization source (ESI), and the multi-reaction detection mode (MRM) was used for content determination. The setup included a desolvation temperature of 450 °C, a source temperature of 150 °C, a desolvation gas flow rate of 800 L/h, a cone gas flow of 150 L/h, and a capillary voltage of 2300 V. The analysis optimized the product ions for two compounds based on the collision-induced dissociation of their precursor ions. Details on the quantitative analysis of ion pairs, fragmentation voltage, and collision energy are provided in Table 5.

### 5.8. Statistical Analysis

The data analysis was conducted using SPSS software (version 19.0; SPSS, Inc. Chicago, IL, USA). Two-way analysis of variance was used to determine the effects of the maternal habitat and salinity and their interaction on all parameters. One-way analysis of variance was used to compare the differences between the treatment groups, and a Duncan test was used as a post hoc test. *P* ≤ 0.05 was considered to display significant differences between treatments. The graphs were prepared using Origin Pro 2017 (OriginLab, Northampton, MA, USA). The column height in the histogram represents the average value of the parameters and the error bar represents the standard deviation from the average value.

## Figures and Tables

**Table 1:** Two-way ANOVA analysis showing the degree of freedom (df), mean squares (MS), F and *p*-values of the effects of salinity and maternal habitat and their interaction on all studied parameters.

Source		Plant Height	Crown Width	Based Stem Diameter	Number of Leaves	Specific Leaf Area	Root Volume
Habitats	Df	1	1	1	1	1	1
	MS	968.1	466.1	0.4	248.5	7536.7	0.1
	F and *p*	196.7 ***	127.6 ***	8.7 **	1.6	30.2 ***	0.28
Salinity	Df	3	3	3	3	3	3
	MS	1391.6	483.2	5.0	16,564.7	8680.9	12.8
	F and *p*	282.8 ***	132.3 ***	125.2 ***	109.7 ***	34.7 ***	70.5 ***
Habitats × Salinity	Df	3	3	3	3	3	3
	MS	15.2	56.4	0.7	139.7	994.6	1.1
	F and *p*	3.1 *	15.4 ***	16.9 ***	0.925	3.9 *	5.8 *

Note:“*” *p* < 0.05; “**” *p* < 0.01; “***” *p* < 0.001.

**Table 2:** Two-way ANOVA analysis showing the degree of freedom (df), mean squares (MS), F and *p*-values of the effects of salinity and maternal habitat and their interaction on all studied parameters.

Source		Root Biomass	Stem Biomass	Leaf Biomass	Glycyrrhizic Acid	Glycyrrhetinic Acid
Habitats	Df	1	1	1	1	1
	MS	0.001	1.5	0.03	1879.7	3487.3
	F and *p*	0.007	141.9 ***	2.9	13.9 **	97.9 ***
Salinity	Df	3	3	3	3	3
	MS	44.25	2.0	3.3	123,888.6	3112.3
	F and *p*	239.1 ***	192.3 ***	308.5 ***	915.3 ***	87.3 ***
Habitats × Salinity	Df	3	3	3	3	3 926.3 ***
	MS	6.0	0.3	0.1	25,350.1	
	F and *p*	32.6 **	30.8 ***	9.3 ***	187.3 ***	

Note: “**” *p* < 0.01; “***” *p* < 0.001.

**Table 3:** Two-way ANOVA analysis showing the degree of freedom (df), mean squares (MS), F and *p*-values of the effects of salinity and maternal habitat and their interaction on all studied parameters.

Source		SOD	POD	CAT	REC	MDA
Habitats	Df	1	1	1	1	1 5.4 *
	MS	2003.1	52,086.7	1011.4	1162.0	
	F and *p*	274.1 ***	304.3 ***	24.4 ***	593.4 ***	
Salinity	Df	3	3	3	3	1
	MS	1382.6	266,270.5	8663.6	3982.4	0.188
	F and *p*	189.2 ***	1555.6 ***	208.9 ***	2033.6 ***	25.5 ***
Habitats × Salinity	Df	3	3	3	3	3
	MS	77.7	67,686.9	414.2	44.7	0.02
	F and *p*	10.6 ***	395.4 ***	10 **	22.8 ***	2.1

Note: “*” *p* < 0.05; “**” *p* < 0.01; “***” *p* < 0.001.

**Table 4:** Two-way ANOVA analysis showing the degree of freedom (df), mean squares (MS), F and *p*-values of the effects of salinity and maternal habitat and their interaction on all studied parameters.

Source	Habitats	Salinity	Habitats × Salinity
	Df	Df	Df
	MS	MS	MS
	F and *p*	F and *p*	F and *p*
Pn	1	3	3
	15.2	883.6	133.9
	2.5	148.9 ***	22.6 ***
Gs	1	3	3
	0.00007	0.1	0.02
	0.1	171.9 ***	24.7 ***
Ci	1	3	3
	40,532.7	42,533.5	97,708.8
	13.8 ***	14.5 ***	33.3 ***
Tr	1	3	3
	9.9	110.0	10.4
	10.9 **	122.0 ***	11.5 ***
F0	1	3	3
	18,278.0	10,808.0	3453.5
	26.1 ***	15.4 ***	4.9 **
Fm	1	3	3
	861.4	101,694.7	2749.9
	0.2	27.7 *	0.7
Fv/Fm	1	3	3
	0.02	0.3	0.003
	37.7 ***	43.5 ***	5.3 **
Y(II)	1	3	3
	0.05	0.09	0.002
	15.6 ***	28.3 ***	0.6
Y(NO)	1	3	3
	0.03	0.01	0.3
	45.4 ***	21.5 ***	0.8
Y(NPQ)	1	3	3
	0.1	0.2	1.3
	39.5 ***	58.54 ***	1.3

Note: “*” *p* < 0.05; “**” *p* < 0.01; “***” *p* < 0.001.

**Table 5:** Optimized mass spectrometry conditions, regression equations, and correlation coefficients for glycyrrhizic acid and glycyrrhetinic acid.

Secondary Metabolite	Parent Ion (m·z^−1^)	Daughter Ion (m·z^−1^)	Ionization Mode	Voltage (V)	Collisional Energy (eV)	Retention Time (min)	Regression Equation	*R* ^2^	Linear Over (ng·mL^−1^)
Glycyrrhizic acid	821.2	350.9 *	–	62	42	2.48	Y = 87.2X − 3.76	0.9992	1.0–978.0
		113.0		62	56				
Glycyrrhetinic acid	469.2	355.1 *	–	100	44	3.81	Y = 136.8X − 122.4	0.9990	1.4–993.2
		409.2		100	46				

Note: “*” represents quantitative ion, *R*^2^ represents correlation coefficient.

## Data Availability

The raw data supporting the conclusions of this article will be made available by the authors on request.
